# The relationship between gender, marital status and depression among Chinese middle-aged and older people: Mediation by subjective well-being and moderation by degree of digitization

**DOI:** 10.3389/fpsyg.2022.923597

**Published:** 2022-10-17

**Authors:** Liangyu Zhao, Kun Zhang, Yan Gao, Zhihao Jia, Suyue Han

**Affiliations:** School of Physical Education, Shandong University, Jinan, Shandong, China

**Keywords:** depression, subjective well-being, degree of digitization, marital status, gender, middle-aged and older people

## Abstract

The purposes of this study were to investigate the effects of gender and marital status on depression among middle-aged and older people in China, and to explore the mediating effect of subjective well-being and the moderating effect of degree of digitization in the relationship between subjective well-being and depression. A total of 15,586 Chinese middle-aged and older people (≥ 45 years old) were included in the study using data from the 2018 China Health and Retirement Longitudinal Survey (CHARLS). *T*-test, ANOVA, hierarchical regression and Bootstrap methods were adopted to test the mediating role of subjective well-being and the moderating role of degree of digitization. The results showed that middle-aged and older women were more likely to suffer from depression than men, and married middle-aged and older people were less likely to be depressed than those who were separated or divorced, widowed and never married. Subjective well-being partially mediated the relationship between gender and depression, and masked the relationship between marital status and depression, and all five dimensions it contains also played a mediating role. Degree of digitization moderated the effect of subjective well-being on depression. Simple slope tests indicated that the effect of subjective well-being on depression increased as degree of digitization increased. In conclusion, to address the mental health problems of middle-aged and older people brought about by the ageing and digital society, we should start by improving their subjective well-being and promoting their integration into the digital society.

## Introduction

The ageing population has become one of the common challenges faced by every country (United Nations, 2019). According to a recent United Nations report, one in six people worldwide is expected to be over the age of 65 (16%) by 2050, and the proportion of older people will double in some regions over the next 30 years, including North Africa, Asia and Latin America (United Nations, 2020). With the highest population and the largest number of older people in the world, China is now one of the countries with the fastest growing ageing population. According to the data of seventh national census of China, the population aged 65 years and above accounted for 13.50% of the total population in 2020, and it is estimated that it will reach 480 million by 2050, and the ageing level will rise to 15.5% (Lin, 2021). As can be seen, China’s older population is increasing dramatically. In this context, the mental health and digital divide of middle-aged and older people (MAOP) have become two hot topics of global and society concern, and whether or not depression is an important evaluation indicator of mental health (Luijendijk et al., 2008; Wu et al., 2018). According to the 2019 report of the World Health Organization, it was estimated that about 350 million people worldwide suffer from depression, and the number of people with depression in China was close to 100 million (Wang et al., 2020). Domestic studies showed that MAOP are one of the populations with the highest prevalence of depression (World Health Organization, 2017). From 1980 to 2000, the prevalence rate of depressive in MAOP in China ranged from about 4.14 to16.55% (Cui et al., 2000; Meng and Tang, 2000), while by 2018, it was found that approximately 23.61% of MAOP surveyed had different degrees of depressive symptoms (Nianwei et al., 2021). Depression not only has an impact on the quality of life, ability of daily living, and cognitive ability of MAOP, but also increases the prevalence of cardiovascular disease (Seligman and Nemeroff, 2015) and suicide rates (Cui, 2015). The World Health Organization classified depression as one of the major factors leading to disability (Smith, 2014).

Among the factors influencing depression in MAOP, gender is one of the most frequently studied variables. The results of several studies showed that there are gender differences in depression, with women being significantly more likely to suffer from depression than men (Hyde et al., 2008; Nianwei et al., 2021). As of 2018, 43.61% of middle-aged and older women in China had depressive symptoms (Ye et al., 2021), that is, more than one-third of middle-aged and older women had depressive symptoms. During the COVID-19 pandemic, women exhibited more severe anxiety, depression, and acute stress symptoms than men (Garcia-Fernandez et al., 2020; Sampson, 2020) and older women may be in greater need of additional mental health support (Kirkman, 2020; Stevens, 2020). In addition, marital status is also a major influencing factor on the physical and mental health of MAOP. Although some studies have shown that married respondents are less likely to be highly happy, they have higher subjective well-being and lower depression than respondents who are divorced, separated, widowed, and never married (Ye et al., 2018; Becchetti and Conzo, 2022). Most studies on depression have treated subjective well-being as a significant predictor variable, whether it can cause a direct or indirect effect. Studies showed that high levels of subjective well-being not only promote physical and mental health and reduce loneliness and depressive symptoms in MAOP, but also can serve as an important indicator of successful aging and contribute to healthy aging (Kleineidam et al., 2019). Lower subjective well-being was the main risk factor for depression in MAOP. Meanwhile, it was found that subjective well-being of MAOP was influenced by factors such as gender, marital status, and education level (Patel et al., 2021). In terms of gender, middle-aged and older women may have lower subjective well-being than men. Compared with MAOP with spouses, those without spouses may have strong feelings of loneliness and lower subjective well-being due to the lack of emotional communication and spiritual comfort (Purol et al., 2021).

In addition, with the advent of the electronic information age, the Internet has penetrated into every aspect of people’s lives, and the popularity of the Internet among MAOP is also increasing. According to the 47th China Internet Development Statistics Report released by the China Internet Network Information Center, as of December 2020, the proportion of older Internet users in China was about 260 million, accounting for 18.4% of the total population. It was predicted that at least a quarter of China’s older population will be internet users by 2030, and Internet will be widely used among the older population by the middle of the 21st century (Zhai et al., 2016). The popularity of the Internet had a great impact on the lifestyles of MAOP, which not only broadened the contact between MAOP and their children, but also diversified their social participation and lifestyle entertainment. Studies showed that the use of the Internet could help alleviate loneliness among MAOP (Song et al., 2019), improve life satisfaction and subjective well-being (Shapira et al., 2007), and reduce risk of depressive symptoms (Chopik, 2016; Xiang et al., 2020). However, some studies found that the use of the Internet increased individuals’ negative emotions such as anxiety and depression (Zhang et al., 2017, 2018), which was not significantly related to well-being.

Although there are many studies on depression in MAOP, most empirical studies mainly focused on the influencing factors of depression, and further investigation of the mechanisms and pathways of the role between gender, marital status and depression in MAOP is lacking. Therefore, by constructing a moderated mediation model, this paper proposed five hypotheses to explore the effects of gender and marital status on depression in MAOP through two pathways: the mediating role of subjective well-being and its five dimensions (life satisfaction, health satisfaction, marital satisfaction, child satisfaction, and air satisfaction) in it, and the moderating role of degree of digitization between subjective well-being and depression. To better construct our research hypotheses, we began the analysis with a comprehensive literature review, summarized the current state of development of depression in MAOP globally and nationally, as well as existing results in terms of gender, marital status, subjective well-being and degree of digitization.

## Theory and hypothesis

A cross-national epidemiological study covering the United States, Canada, France, Italy, New Zealand, Puerto Rico, West Germany, Lebanon, Taiwan, and South Korea showed that women were significantly more likely to suffer from depression than men (Weissman et al., 1996). Despite the influence of regional culture, most countries reported that in adulthood, the number of women with depression was about twice that of men (Nolen-Hoeksema, 2001; Lucht et al., 2003; Simo-Noguera et al., 2015; Iranpour et al., 2022). And a study during the COVID-19 pandemic showed that older women were 2.07 times more likely than men to report depressive symptoms and more likely to report anxiety and loneliness (Reppas-Rindlisbacher et al., 2022). According to emotional intensity theory, women reported higher levels of positive and negative emotions, had higher emotional intensity and reacted more strongly to the same events than men (Brehm, 1999). In reality, women were more negatively affected than men, had higher sensitivity to positive or negative events in life, and needed to take more time to recover to their previous level of health after experiencing negative shocks (Becchetti and Conzo, 2022). Therefore, depressive symptoms are easily triggered during prolonged negative emotions (Bullinger, 1989; Hewitt et al., 1992; Marques and Lima, 2011). Domestic studies showed that age has an “inverted U” parabolic shape on depressive symptoms in women. The peak age of onset of depression in women is 52 years old. With the increase of age, the symptoms of depression first aggravate and then alleviate. The peak age of onset is 50–60 years old (Li and Ma, 2017), that is, middle-aged and older women were more likely to have depressive symptoms (Wu et al., 2019). The study found that married women showed more mental symptoms, and their positive mental health level and perceived health status were lower than those of men (Shek, 1995). In most countries, people who were separated or divorced were markedly more likely to suffer from major depression than married people. Middle-aged and older men living alone showed more severe loneliness, depressive symptoms and suicidal ideation than women of the same age (Kim, 2017; Park and Choi, 2020), and widowed MAOP would have a stronger sense of loneliness (Ye et al., 2018; Van Tilburg and Suanet, 2019; Pan and Liu, 2021). The subjective well-being of MAOP who had never been married or had no children was not significantly different from that of married peers (Van Tilburg and Suanet, 2019). Subjective well-being was negatively and dramatically associated with depression. Therefore, based on the above research, we proposed the following hypotheses:

*Hypothesis 1*: Middle-aged and older women are more likely to suffer from depression than men.

*Hypothesis 2*: Married MAOP are less likely to suffer from depression than those who are separated, divorced, widowed, and never married.

Subjective well-being referred to a comprehensive and integrated assessment of the quality of life by individuals based on their own standards (Diener et al., 1999). It was an important indicator to measure people’s quality of life and mental health (Oswald and Wu, 2010). Currently, instruments such as the Subjective Well-being Scale (Pavot and Diener, 1993), the Subjective Well-being Questionnaire for the Older (Chen, 2009), and the General Well-being Scale (Fazio, 1977) have been used to measure subjective well-being. According to the 2018 national baseline questionnaire of the China Health and Retirement Longitudinal Survey (CHARLS), the measures of subjective well-being in this study included “life satisfaction,” “health satisfaction,” “marriage satisfaction,” “children satisfaction” and “air satisfaction.” According to psychoanalytic theory, the main source of individual’s happiness was their instinctive feelings, especially the great satisfaction of sexual instincts. Needs satisfaction theory suggested that subjective well-being depends on the satisfaction of one’s physiological and psychological needs. Positive feelings were most associated with social and respect needs, and negative feelings were most associated with basic, respect and autonomy needs. Social need fulfillment exceeded an individual’s fulfillment of their own needs in predicting subjective well-being (Tay and Diener, 2011). Therefore, considering the global gender inequality (United Nations Development Programme, 2016), women have lower subjective well-being than men on average (Richardson, 2021). However, it was also shown that there was no significant difference in subjective well-being between older men and women (Liu, 2011). Emotional support theory suggested that social networks could reduce stress and increase personal well-being through connections with family, relatives, friends and neighbors (Leung et al., 2011), while MAOP could have their social networks shrink due to retirement, declining health and the deaths of family members and friends (Bruce, 2002). In these circumstances, spouses often become the primary and important emotional supporter. Thus, MAOP with a spouse had significantly higher subjective well-being than those who were widowed (Lucas et al., 2003), divorced and separated (Perelli-Harris et al., 2019). Due to the decline of physical health and social support, MAOP may be more prone to strong feelings of loneliness and lower subjective well-being, which could lead to depressive symptoms. Therefore, it is important to investigate how subjective well-being maintains the psychological health and improves the quality of life of MAOP. Based on the above study, we made the following hypotheses:

*Hypothesis 3*: Subjective well-being of MAOP plays a mediating effect between gender and depression.

*Hypothesis 4*: Subjective well-being of MAOP plays a mediating effect between marital status and depression.

In 1999, the World Health Organization introduced the slogan “active aging” on the basis of “healthy aging,” which aimed to prolong healthy life expectancy and improved the quality of life. In order to realize “active aging,” the second World Assembly on Ageing proposed that older people should learn information technology to integrate into the digital society more quickly. Nowadays, digital society and the ageing population are two parallel trends, and the integration of older people and digitalization has gradually become a hot topic of general concern worldwide. The popularity of the Internet had a great impact on the lifestyle of MAOP. It not only expanded the mode of interaction with relatives and friends, but also improved their social adaptability (Wu and Peng, 2018). According to reinforcement theory, positive feedback on the consequences of people’s behavior helped people to reinforce their behaviour (Skinner, 1958; Huang, 2012). The use of the Internet enriched the social interactions of MAOP, enhanced their social support, and increased their subjective well-being, thus accelerating the integration between them and digital society (Kraut et al., 2002). Therefore, Internet was a technological tool that enhances subjective well-being of MAOP (Pantic, 2014). Meanwhile, the use of Internet could help them build new friendships, maintain social engagement (Chen and Schulz, 2016), and reduce loneliness (Fokkema and Knipscheer, 2007; Khalaila and Vitman-Schorr, 2018). There was a significant positive association between loneliness and depression (Domenech-Abella et al., 2017). Consequently, the use of the Internet could improve the mental health of MAOP (Heo et al., 2015) and reduce the possibility of depression (Cotten et al., 2012; Reneland-Forsman, 2018; Hajek and Koenig, 2021). Based on the above research, we made the following hypothesis:

*Hypothesis 5*: Degree of digitization plays a moderating role between subjective well-being and depression in MAOP.

**Figure 1:**
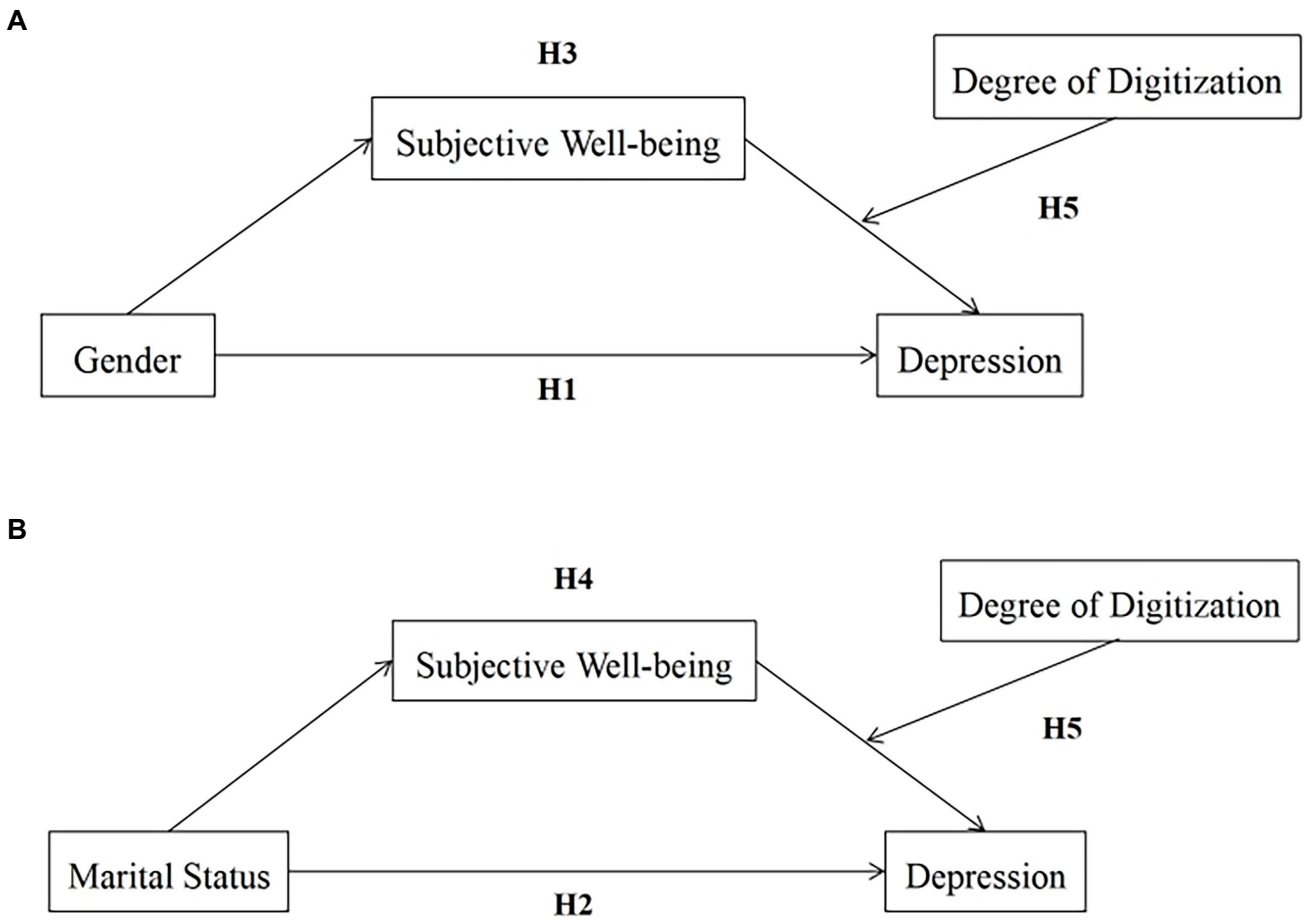
The hypothesized model: **(A)** Hypothetical model between sex and depression; **(B)** Hypothetical model between marital status and depression.

In summary, based on the above five research hypotheses, the following model were constructed (see Figure 1).

## Materials and methods

### Data source

This paper uses the 2018 national baseline survey data of China Health and Retirement Longitudinal Survey (CHARLS) (Zhao et al., 2020). CHARLS, hosted by the National Development Institute of Peking University, aims to collect a set of high-quality micro data representing the MAOP aged 45 and above and their families to analyze China’s ageing population. The CHALRS questionnaire was designed with reference to international experience from the Health and Retirement Survey (HRS) in the U.S., the Elderly Longitudinal Survey (ELSA) in the U.K., and Survey of Health, Aging and Retirement in Europe (SHARE). The questionnaire adopted multi-stage PPS sampling and used the electronic mapping software (CHALRS-GIS) technology pioneered by CHARLS to create a village-level sampling frame using the map method to list all dwelling units in a community, and then randomly selected a certain number of dwelling units. CHARLS was approved by the Biomedical Ethics Committee of Peking University (IRB00001052-11015), and each subject signed an informed consent form. The 2018 CHARLS survey interviews involved 17708 individuals from 10257 families, and was publicly released on September 24, 2020. Cross-sectional data were analyzed in this study, and MAOP aged 45 years and older were selected for the study, which ultimately included 15,586 individuals after removing a large number of missing data. The CHARLS data was used in this study not only because it has the required variable settings for the study, but also because it is strictly based on a sampling method and a large sample of MAOP, which made the results more representative.

### Variable description

#### Variable selection

This paper first studied the differences in depression among MAOP by gender and marital status, and then explored the mediating effect of subjective well-being and the moderating effect of degree of digitization between subjective well-being and depression. Thus, the dependent variable is depression, and the independent variables are sex and marital status. We converted sex to a dummy variable, denoted by 0 (female) and 1 (male), and marital status to a multicategorical variable, categorized as married, separated or divorced, widowed, and never married. The mediating variable is subjective well-being, the moderating variable is degree of digitization, and the control variables are age, address and education level. Both independent variables are definite class variables, the mediating and dependent variables are quantitative variables, and the mediating variable is a definite order variable. The variables are assigned as shown in Table 1.

**Table 1:** Variable assignment.

Variables	Original question	Codes
Age	What’s your date of birth on ID card or Household register?	1 = 45–54 years old; 2 = 55–64 years old; 3 = 65–74 years old; 4 = 75 years old and above
Address	What was the type of your address?	1 = The center of city/town; 2 = Combination zone between urban and rural areas; 3 = Village; 4 = Special area
Education	What’s the highest level of education your have now (not including adult education)?	1 = No formal education (illiterate) or did not finish primary school; 2 = Sishu/home or Elementary school; 3 = Middle school; 4 = High or vocational school; 5 = Two-/Three-Year College/Associate or Four-Year College/Bachelor’s degree and above
Sex	Interviewer record the Respondent’s gender.	1 = Male; 0 = Female
Marital status	What is your marital status?	1 = Married; 2 = Separated or divorced; 3 = Widowed; 4 = Never married
Degree of digitization	(1) Have you used the Internet in the last month? (2) How often in the last month did you use the Internet? (3) Do you use mobile payments, such as Alipay and WeChat pay? (4) Do you use WeChat? (5) Do you post WeChat moments?	0 = No; 1 = Very weak; 2 = Weak; 3 = A little weak; 4 = general; 5 = A little strong; 6 = Strong; 7 = Very strong
Life satisfaction	Please think about your life-as-a-whole. How satisfied are you with it?	1 = Completely satisfied; 2 = Very satisfied; 3 = Somewhat satisfied; 4 = Not very satisfied; 5 = Not at all satisfied
Health satisfaction	How satisfied are you with your health?	1 = Completely satisfied; 2 = Very satisfied; 3 = Somewhat satisfied; 4 = Not very satisfied; 5 = Not at all satisfied
Marriage satisfaction	How satisfied are you with your marriage (relationship with spouse)?	0 = No spouse now; 1 = Completely satisfied; 2 = Very satisfied; 3 = Somewhat satisfied; 4 = Not very satisfied; 5 = Not at all satisfied
Child satisfaction	How satisfied are you with your relationship with children?	0 = No child now; 1 = Completely satisfied; 2 = Very satisfied; 3 = Somewhat satisfied; 4 = Not very satisfied; 5 = Not at all satisfied
Air satisfaction	How satisfied are you with the air quality this year?	1 = Completely satisfied; 2 = Very satisfied; 3 = Somewhat satisfied; 4 = Not very satisfied; 5 = Not at all satisfied

#### Depression

In this paper, the Epidemiological Survey Depression Scale (CES-D-10) was used for measurement. The CES-D 10-item short scale overcomes the problems of long response time and high refusal rate of respondents during the measurement of the original CES-Delderly 20-item scale. The scale has been widely used and has good reliability and validity in Chinese population (Huang et al., 2015). The Cronbach’s alpha of this study was 0.805, and the reliability and validity were good. CES-D-10 consists of 10 questions about the participant’s past week experience, with consistent answers for each entry, including 0 points for little or none (<1d), 1 point for not too much (1-2d), 2 points for sometimes or half of the time (3-4d), and 3 points for most of the time (5-7d), with reverse scoring used for entries 5 and 8. The summary score ranges from 0 to 30, with higher scores associated with stronger depressive symptoms.

#### Subjective well-being

Based on the satisfaction questions included in the CHARLS questionnaire, this paper used five of these items in the questionnaire to measure subjective well-being, namely “DC028 How satisfied are you with your life-as-a-whole?,” “DC042_W3 How satisfied are you with your health?,” “DC043_W3 How satisfied are you with your marriage (relationship with spouse)?,” “DC044_W3 How satisfied are you with your relationship with children?” and “DC046_W4 How satisfied are you with the air quality this year?” “Life satisfaction,” “health satisfaction” and “air satisfaction” are measured on a Likert five-level scale, including “not satisfied at all “(1 point), “not very satisfied” (2 points), “quite satisfied” (3 points), “very satisfied” (4 points) and “extremely satisfied” (5 points). We, respectively, added “No spouse now” and “No child now” (0 point) to “marital satisfaction.” The Cronbach’s alpha for the five items was 0.653, which was higher than the minimum of 0.6, and is therefore reliable for measuring subjective well-being (Kline, 2011). We used a five-item summation method with scores ranging from 3 to 25, the higher the score, the greater the subjective well-being.

#### Degree of digitization

Five questions in CHARLS were used to measure degree of digitization. They are “DA056_10 Have you used the Internet in the last month?” “DA057_10 How often in the last month did you use the Internet?” “DA056_W4_2 Do you use mobile payments, such as Alipay and WeChat pay?” DA056_W4_3 Do you use WeChat?” “DA056_W4_3 Do you use WeChat?” and “DA056_W4_4 Do you post WeChat moments?” The answers to items 1, 3, 4 and 5 are “yes” (1 point) and “no” (0 point). The answers to Item 2 include “almost every day” (3 points), “almost every week” (2 points) and “not often” (1 point). The scores of the five answers were added together. The summary score ranges from 0 to 7, with the higher the score, the higher the degree of digitization.

### Statistical analysis

SPSS 26.0 and R Statistical language 4.1.3 were used to analyze the data. First, we used independent samples t-tests, one-way ANOVA and chi-square tests to explore differences in depression, subjective well-being, and degree of digitization by sex and by marital status. A value of p of less than 0.05 indicated a significant difference. Then, mediating and moderating effects were tested by hierarchical regression. We examined the mediating effect of subjective well-being using PROCESS v3.5, which was indicated if the 95% confidence interval did not include 0. Second, we examined the moderating effect of the degree of digitization, using the interaction term (subjective well-being × degree of digitization) to test whether it was significant, and if the interaction term showed significance, then a moderating effect of the degree of digitization was demonstrated. Finally, we performed a simple slope analysis and plotted the graph using R statistical language and ggplot2.

## Results

### Common method deviation test

Because the data in this study were obtained from subjects’ self-reports, the data results are susceptible to common method bias. We used the Harman one-way ANOVA test for the presence of common method bias before data analysis (Podsakoff et al., 2003), and an exploratory factor analysis was performed on all topics of the study variables. The results showed that there were four factors with eigenvalues greater than one, and the explained variance of the first principal factor was 24.796%, which was below the critical value of 40%, indicating that there was no significant common method bias.

### Descriptive statistics and analysis of variance

**Figure 2:**
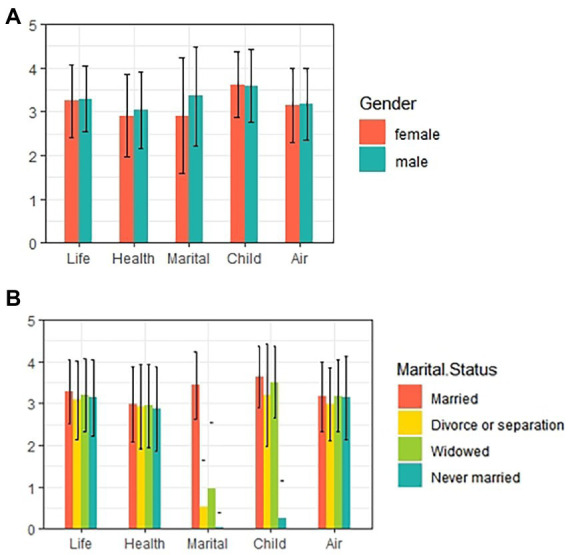
The distribution of five dimensions of subjective well-being: **(A)** Distribution in sex; **(B)** Distribution in marital status.

A total of 15,586 MAOP were included in this study. Among them, women (51.06%) were slightly more than men (48.94%). The majority of MAOP have a low level of education, with 38.77% of them indicating that they had not received education (illiterate) or not finished primary school. Most of MAOP (87.63%) were currently married and their spouses were still alive. The degree of digitization of them was low, and 85.08% of them did not surf the Internet. The overall subjective well-being of MAOP was good, with an average score of 16.116. The differential analysis of variables was given in Table 2. As shown in Table 2, MAOP of different sex showed significant differences for depression [*t*(15586) = 21.485, *p* < 0.01], and middle-aged and older women (*M* = 9.50, *SD* = 6.85) were more depressed than man (*M* = 7.30, *SD* = 5.88). Hypothesis 1 held. MAOP by marital status also showed significant differences for depression [*F*(3) = 73.025, *p* < 0.01], and *post hoc* multiple tests indicated that married MAOP (*M* = 8.14, *SD* = 6.32) were less depressed than those who were separated or divorced (*M* = 9.74, *SD* = 7.02), widowed (*M* = 10.51, *SD* = 7.27) and never married (*M* = 11.19, *SD* = 7.22), and Hypothesis 2 held. Both sex and marital status showed significant differences on subjective well-being among MAOP [*t*(15586) = −13.087, *p* < 0.01; *F*(3) = 719.453, *p* < 0.01]. Male (*M* = 16.43, *SD* = 2.86) had significantly higher subjective well-being than female (*M* = 15.82, *SD* = 3.01), and married MAOP (*M* = 16.48, *SD* = 2.72) had significantly higher subjective well-being than separated or divorced (*M* = 12.70, *SD* = 3.07), widowed (*M* = 13.82, *SD* = 3.13) and never-married (*M* = 9.45, *SD* = 2.28). Because sex, marital status and the five dimensions included in subjective well-being are categorical variables, we conducted chi-square tests before testing for mediating effects and plotted the distribution of responses for the five dimensions using R-4.1.3 and ggplot2 (as shown in Figure 2). As shown in Table 3, significant differences (*p* < 0.01) were observed across sex and marital status for life satisfaction, health satisfaction, marital satisfaction, child satisfaction, and air satisfaction.

**Table 2:** A differential analysis of sex, marital status, subjective well-being and depression (*n* = 15,586).

		Depression			Subjective well-being		
		*x̅* ± s	*t*	*p*	*x̅* ± s	*t*	*p*
Sex	Male	7.30 ± 5.88	21.485^**^	0.000	16.43 ± 2.86	−13.087^**^	0.000
	Female	9.50 ± 6.85			15.82 ± 3.01		
		*x̅* ± s	*F*	*p*	*x̅* ± s	*F*	*p*
Marital status	Married	8.14 ± 6.32	73.025^**^	0.000	16.48 ± 2.72	719.453^**^	0.000
	Separated or divorced	9.74 ± 7.02			12.70 ± 3.07		
	Widowed	10.51 ± 7.27			13.82 ± 3.13		
	Never married	11.19 ± 7.22			9.45 ± 2.28		

* *p* < 0.05;

** *p* < 0.01.

**Table 3:** The chi-square test between sex, marital status and the five dimensions of subjective well-being (*n* = 15,586).

	Sex			Marital status		
	*χ* ^2^	*p*	Lambda	*χ* ^2^	*p*	Lambda
Life satisfaction	79.797	0.000^**^	0.000	90.378	0.000^**^	0.000
Health satisfaction	127.568	0.000^**^	0.000	49.512	0.000^**^	0.000
Marriage satisfaction	660.362	0.000^**^	0.024	10849.646	0.000^**^	0.126
Child satisfaction	80.100	0.000^**^	0.000	7835.912	0.000^**^	0.010
Air satisfaction	31.215	0.000^**^	0.000	45.498	0.000^**^	0.000

* *p* < 0.05;

** *p* < 0.01.

### The test of mediating effects of subjective well-being and its five dimensions

Because sex was a dichotomous variable in this study and both the mediating and dependent variables were continuous variables, a hierarchical regression was used to test for mediating effects. As shown in Table 4, the first time control variables (age, address, education level) and sex were added, and the results showed that sex had a significant effect on depression in MAOP (*β* = −0.136, *p* < 0.01). The second time, the dependent variable was changed to subjective well-being, and the results showed that sex also had a significant effect on subjective well-being (*β* = 0.644, *p* < 0.01); the third time, subjective well-being was added to the first time, and the results showed that subjective well-being also had a significant effect on depression (*β* = 0.879, *p* < 0.01). Therefore, subjective well-being partially mediated the effect between sex and depression, and Hypothesis 3 held. We used relative and overall mediators to analysis the mediating role of subjective well-being between marital status and depression (Fang et al., 2017). Model 4 in PROCESS v3.5 was used, with different marital status coded as dummy variables, bootstrap set to 5000, confidence interval set to 95%, and control variables, independent variables, mediating variable, and dependent variable were sequentially placed in the box to obtain the analysis results. As shown in Table 5, using the married group as a reference, the relative indirect effect of subjective well-being between the separated or divorced group and depression was 3.517, with a 95% bootstrap confidence interval of [3.144, 3.895], excluding “0,” indicating a significant relative indirect effect. After adding the subjective well-being, the relative direct effect of separation or divorce on depression was −1.292, with a 95% bootstrap confidence interval of [−2.045, −0.539], excluding “0,” indicating that the relative direct effect was also significant, but the two effect values were different, suggesting that subjective well-being played a masking role between the separation or divorce and depression. The relative indirect effect of subjective well-being on depression in the widowed group was 2.553, with a 95% bootstrap confidence interval of (2.371, 2.742), excluding “0,” indicating a significant relative indirect effect. After the inclusion of mediating variables, the relative direct effect of widowhood on depression was −0.643, with a 95% bootstrap confidence interval of (−1.007, −0.279), excluding “0,” indicating that the relative direct effect was also significant, but the two effect values were different, suggesting that subjective well-being played a masking role between widowhood and depression. The relative indirect effect of never married group on depression through subjective well-being was 6.643, with a 95% Bootstrap confidence interval of (6.100, 7.194), excluding “0,” indicating a significant relative indirect effect. After adding subjective well-being, the relative direct effect of never married on depression was −4.376, with a 95% bootstrap confidence interval of (−5.975, −2.777), excluding “0,” indicating that the relative direct effect was also significant, but the effect values were also different, indicating that subjective well-being also played a masking role between never married and depression, and Hypothesis 4 held.

**Table 4:** The test of the mediating effect of subjective well-being between sex and depression (total and direct effects).

Variables	Depression				Subjective well-being				Depression			
	*B*	*SE*	*t*	*β*	*B*	*SE*	*t*	*β*	*B*	*SE*	*t*	*β*
Age	0.202^**^	0.054	3.732	0.030	−0.202^**^	0.025	−7.951	−0.060	0.025	0.050	0.502	0.004
Address	0.705^**^	0.066	10.750	0.089	0.082^**^	0.031	2.683	0.023	0.777^**^	0.060	13.005	0.099
Education	−0.900^**^	0.050	−17.961	−0.157	−0.021	0.023	−0.902	−0.008	−0.919^**^	0.046	−20.115	−0.160
Sex	−1.766^**^	0.104	−16.942	−0.136	0.644^**^	0.049	13.190	0.109	−1.200^**^	0.096	−12.561	−0.092
Subjective well-being									−0.879^**^	0.016	−56.399	−0.400
*R* ^2^	0.072				0.016				0.229			
*F*	303.027^**^				61.657^**^				928.061^**^			

* *p* < 0.05;

** *p* < 0.01 and

*N* = 15586.

**Table 5:** The mediating effects test of subjective well-being (direct and indirect effects).

Mediating effect path	Effect	Boot LLCI	Boot ULCI
Sex→Subjective well-being→Depression	−0.566^a^	−0.656	−0.479
Sex→Depression	−1.200^a^	−1.387	−1.013
*Married group as control*			
Separated or divorced→Subjective well-being→Depression	3.517^a^	3.140	3.900
Separated or divorced→Depression	−1.292^a^	−2.017	−0.566
Widowed→Subjective well-being→Depression	2.553^a^	2.371	2.738
Widowed→Depression	−0.643^a^	−0.965	−0.321
Never married→Subjective well-being→Depression	6.643^a^	6.097	7.195
Never married→Depression	−4.376^a^	−5.702	−3.050

LLCI, lower 95% level confidence interval; ULCI, upper 95% level confidence interval. a indicates that the direct effect or indirect effect is significant (*p* < 0.01). *N* = 15586.

To further investigate which dimensions of subjective well-being played a mediating role, we conducted chi-square tests between the independent variables and the five dimensions, and ANOVA between the five dimensions and depression. As shown in Table 6, significant differences (*p* < 0.01) were found across sex and marital status for life satisfaction, health satisfaction, marital satisfaction, child satisfaction, and air satisfaction. Because all five dimensions are fixed-order variables, the effect sizes were looked at according to Lambda indicators. The Lambda values between the variables were all less than 0.2, and the magnitude of the differences was small. The results of the ANOVA between the five dimensions and depression showed that all five dimensions showed significant differences (*p* < 0.01) for depression, with the *η*^2^ for life satisfaction and health satisfaction for depression being greater than 0.14, which is a large difference. Thus, life satisfaction, health satisfaction, marital satisfaction, child satisfaction and air satisfaction all played a mediating role.

**Table 6:** ANOVA between the five dimensions of subjective well-being included and depression.

	Depression		
	*F*	*p*	Eta^2^
Life satisfaction	910.897	0.000^**^	0.190
Health satisfaction	891.328	0.000^**^	0.186
Marriage satisfaction	305.316	0.000^**^	0.089
Child satisfaction	168.921	0.000^**^	0.051
Air satisfaction	56.04	0.000^**^	0.014

* *p* < 0.05;

** *p* < 0.01.

### The test of the moderating effect of the degree of digitization

**Figure 3:**
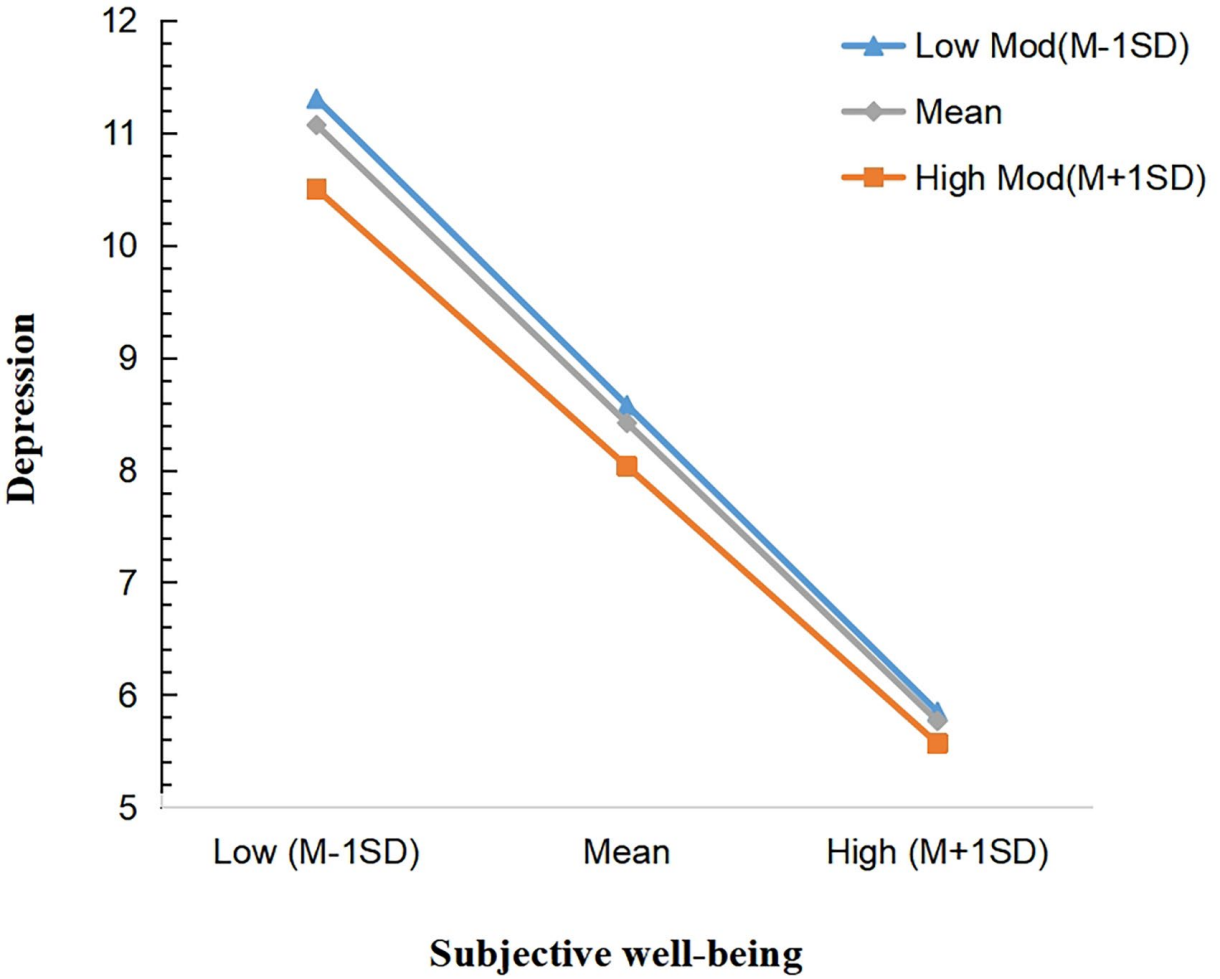
Simple slope analysis. The blue line represents a low level of degree of digitization and the orange line represents a high level of degree of digitization.

Model 1 in the PROCESS was used to test the moderating effect of the degree of digitization. Because both subjective well-being and degree of digitization are quantitative variables, they were centralized. The moderating effect was tested controlling for age, address, and education level (Table 7). The results showed that the product of subjective well-being and degree of digitization had a significant predictive effect on depression when degree of digitization was put into the model (*t* = 3.861, *p* < 0.01), indicating that degree of digitization played a moderating role in the prediction of depression by subjective well-being and that Hypothesis 5 was valid. To further examine the nature of the moderating effect, we conducted a simple slope analysis, as shown in Figure 3. The negative predictive effect of subjective well-being on depression was higher when MAOP were less digital (M-1SD), simple slope = −0.952, *p* < 0.01, and lower when MAOP were more digital (M + 1SD), simple slope = −0.836, *p* < 0.01, suggesting that the predictive effect of subjective well-being on depression diminished as the degree of digitization level increased.

**Table 7:** The moderating effect test of degree of digitization.

Variables	Model 1				Model 2			
	*B*	*SE*	*t*	*β*	*B*	*SE*	*t*	*β*
Age	−0.122^*^	0.050	−2.423	−0.018	−0.125^*^	0.050	−2.493	−0.018
Address	0.618^**^	0.060	10.254	0.078	0.611^**^	0.060	10.134	0.077
Education	−0.976^**^	0.046	−21.2	−0.170	−0.977^**^	0.046	−21.226	−0.17
Subjective well-being	−2.658^**^	0.046	−57.921	−0.410	−2.65^**^	0.046	−57.73	−0.408
Degree of digitization	−0.382^**^	0.051	−7.506	−0.059	−0.385^**^	0.051	−7.561	−0.059
Subjective well-being × degree of digitization					0.185^**^	0.048	3.861	0.027
Δ*R*^2^	0.224				0.001			
*F*	901.996^**^				14.908^**^			

Δ*R*^2^ refers to the added value of the proportion of the variation in the dependent variable that is explained by the regression equation as the new independent variable continues to enter the model.

* *p* < 0.05;

** *p* < 0.01.

## Discussion

### Gender, marital status and depression in MAOP

This study confirmed that there was a significant correlation between gender, marital status and depression in MAOP, with women being more likely to be depressed than men, and the married being less likely to be depressed than those who were separated or divorced, widowed, and never married, which is consistent with previous studies (Cheng and Jiang, 2017; Perelli-Harris et al., 2019; Reppas-Rindlisbacher et al., 2022). On the one hand, this may be due to the influenced by the differences of Chinese traditional culture, most MAOP live in the division of labor mode of “the woman stayed at home and the man earned the money.” Compared to men, women are required to perform more domestic work and bear the stress of childbirth and childcare (Yang, 2014), and stress is a significant predictor of depression. In addition, the findings of this study are also consistent with the role conflict problem for women in role theory (Guoan, 2009). A study on feminism in China showed that when women encounter the problem of dual role conflict between family and work, women’s work role often has to be subordinated to their family role, resulting in losing job and promotion opportunities, lowering their social status and sense of self-worth and making them more likely to become depressed (Xiong, 2010). On the other hand, women need to spend more time than men in alleviating the negative impact (Becchetti and Conzo, 2022). Therefore, middle-aged and older women are significantly more likely to suffer from depression than men. Previous studies showed that MAOP were at risk of shrinking social networks and social isolation after experiencing retirement or unemployment, and that the care and support of their spouses was an important resource to alleviate their psychological fallout and loneliness and reduced the probability of depression, so MAOP with spouses were less likely to suffer from depression than the divorced and the widowed (Eun and Hong, 2015; Cheng and Jiang, 2017), and our findings are consistent with this. For the separated or divorced, they feel little support in an unhappy marital relationship. The widowed are immersed in sadness and pain for a long time, and they all show more psychological symptoms such as depression and anxiety (Cheng and Jiang, 2017). Unmarried MAOP also experience strong feelings of loneliness and depressive symptoms due to the absence of a spouse and children. In the current COVID-19 pandemic, frequent fear and loneliness due to isolation make people experience more severe stress, anxiety and depression symptoms, especially women and older people (Gebhard et al., 2020; Liu et al., 2020; Mauvais-Jarvis et al., 2020) and therefore more social support is needed for them to maintain their mental health.

### The mediating role of subjective well-being

The results suggest that subjective well-being partially mediates the relationship between gender and depression, i.e., gender not only has a direct effect on depression but can also have an indirect effect on it through the mediating role of subjective well-being, which is consistent with the majority of previous studies (Senik, 2017; Chen et al., 2019). Previous studies showed that gender was an important factor in subjective well-being, especially after middle age, when the subjective well-being gap between the sexes became more pronounced (Meisenberg and Woodley, 2015; Senik, 2017). In this study, middle-aged and older men had higher subjective well-being than women, consistent with previous findings (Richardson, 2021). A study that included 95 countries showed that subjective well-being was higher for women in 50 countries and for men in 45 countries, with women having higher subjective well-being than men on average, which correlated with the level of gender equality in the country (Arrosa and Gandelman, 2016). In recent years, the status of women in China has gradually increased, as has the degree of gender equality, and women are trying to move away from the traditional female role of “merely assisting their husbands and raising their children” and move more towards their own careers. However, some studies found that high female employment rates or adherence to the value system of female employment may reduce women’s well-being and subjective well-being (Meisenberg and Woodley, 2015), which may explain the findings of this study. Some studies also showed that there was no significant difference in subjective well-being between MAOP of different genders (Liu, 2011). Thus, the relationship between gender and subjective well-being is influenced by a number of factors. In this study, subjective well-being played a masking role between marital status and depression, that is, the effect of marital status on depression became stronger after the introduction of subjective well-being. This suggests that people’s subjective well-being is an important antecedent of depression in different marital status. To our knowledge, this is the first study to find a masking effect of subjective well-being between marital status and depression. Numerous previous studies showed that marital status had a significant impact on subjective well-being (DeMaris and Oates, 2022). Married or cohabiting MAOP had higher subjective well-being than those who were widowed, divorced or living alone (Cheng and Yan, 2021; Jing et al., 2021), with good interpersonal relationships and family support being the main positive factors (Xing et al., 2010). However, some studies found that no significant difference in subjective well-being between MAOP who live alone, are unmarried, and have no children, and those who are married or cohabiting (Koropeckyj-Cox, 1998; Matsuura and Ma, 2021). Therefore, the relationship between marital status and subjective well-being is also somewhat controversial. In this paper, subjective well-being had a significant negative effect on depression, which is consistent with previous studies (Wood and Joseph, 2010; Chen et al., 2019). It has been suggested that an individual’s physical and mental health is closely related to subjective well-being, and that the stronger the subjective well-being, the more positive the experience and the less affected by depression (Zhang et al., 2020). A longitudinal study found that people with low positive well-being were more than twice as likely to be depressed 10 years later than normal (Wood and Joseph, 2010). Therefore, improving the subjective well-being of MAOP is an effective means of reducing and preventing depression.

### Moderating effect of degree of digitization

This study confirmed that the degree of digitization plays a moderating role between subjective well-being and depression in MAOP, and that the effect of subjective well-being on depression increases with the increase of the degree of digitization, which is consistent with previous studies (Song et al., 2019; Jiang and Chen, 2021). This is mainly because on the one hand, with the continuous optimisation and upgrading of the Internet infrastructure and the deep integration of transport, healthcare and financial sectors with the Internet, the lives of MAOP are becoming more convenient and intelligent, which has improves their quality of life and subjective well-being (Yuqi and Tamura, 2020). On the other hand, the use of the Internet may enable MAOP to maintain close intergenerational ties and increase their sense of belonging to the community, thus contributing to their subjective well-being (Li and Zhou, 2021). Studies in some other countries also found that the use of the Internet has a positive impact on the subjective well-being of MAOP and helps to reduce their feelings of loneliness and depression (Jin and Zhao, 2019; Song et al., 2019; Li and Zhou, 2021). According to data from the 2016 Social Tracking Survey of Older People in China, watching news, chatting, and watching movies and dramas are the top functions most frequently used by older people. However, studies found that Internet use for learning is positively associated with subjective well-being, but Internet use mainly for gaming and leisure is positively associated with perceived stress, depression and anxiety (Prizant-Passal et al., 2016; Paez et al., 2020). Therefore, we suggest that MAOP should control the amount of time they spend online and use the Internet more for chatting, reading news and studying, and less for playing games and relaxing.

### Strengths and limitations

This study had several strengths. First, to our knowledge, this study was the first to explore the mediating effect of subjective well-being between gender and depression, between marital status and depression, and the moderating effect of degree of digitization between subjective well-being and depression in MAOP. Our findings added information for effective prevention and intervention of depression in MAOP. Secondly, this study used a nationally representative sample, which better represented the real situation of MAOP in China and enabled the results to be generalised to MAOP across the country. There were some limitations in this study. Firstly, this study was a cross-sectional study. Despite the sample size of this study was both large and broad, it was difficult to dynamically reflect the relationship between sex or marital status and depression in MAOP. The findings of this study verified the differences and correlations between variables, but could not lead to a causal relationship between variables. Therefore, in future studies, longitudinal data can be used to examine the interaction between variables and whether sex and marital status have a persistent effect on depression, so as to better understand the causes of depression in MAOP. Secondly, the data included in this study were collected in a self-report format, and the use of self-report measures alone predisposes the findings to bias from recall and social expectations. Future studies should use multiple sources of information (such as children, spouses, friends, etc.) to collect data to enhance the reliability of the research results. Finally, in this study, subjective well-being played a partial mediating effect between gender and depression and a masking role between marital status and depression. Therefore, other mediating variables and moderating variables need to be considered in future studies to find more factors affecting depression, so as to prevent and reduce depression in the middle-aged and older.

## Conclusion

This study focused on the relationship between sex, marital status and depression in MAOP, as well as the mediating role of subjective well-being and the moderating role of degree of digitization in the relationship between subjective well-being and depression. The results showed that sex and marital status had significant effects on depression in MAOP, and that sex and marital status could have an effect on depression through subjective well-being. The results also confirmed that the degree of digitization could moderate the effect of subjective well-being on depression, and the higher the degree of digitization, the stronger the effect of subjective well-being on depression, which was generally consistent with the results of earlier studies. Our study helps to expand the factors influencing depression in MAOP and reveal its mechanism of action, especially the effect of degree of digitization. It also helps policy makers and mental health therapists to better address depression among MAOP in China, and has important theoretical and practical guidance for preventing and reducing depression among MAOP.

## Data availability statement

Publicly available datasets were analyzed in this study. This data can be found at: http://charls.pku.edu.cn/.

## Author contributions

LZ: conceptualization, methodology, data analysis, writing-original draft, and visualization. YG: data analysis, methodology, formal analysis, supervision, writing-review and editing, project administration, and funding acquisition. KZ: data analysis and visualization. ZJ: data analysis and formal analysis. SH: conceptualization. All authors contributed to the article and approved the submitted version.

## Funding

This research was funded by the National Social Science Foundation of China (grant number: 21BTY054), China Postdoctoral Science Foundation (grant number: 2017 M622169), and Future Project for Youth Scholar of Shandong University (grant number: 2017WLJH17). The content of the paper is solely the responsibility of the authors and does not necessarily represent the official views of the funders.

## Conflict of interest

The authors declare that the research was conducted in the absence of any commercial or financial relationships that could be construed as a potential conflict of interest.

## Publisher’s note

All claims expressed in this article are solely those of the authors and do not necessarily represent those of their affiliated organizations, or those of the publisher, the editors and the reviewers. Any product that may be evaluated in this article, or claim that may be made by its manufacturer, is not guaranteed or endorsed by the publisher.

## Abbreviations

MAOP: middle-aged and older people.
